# The Dietary Flavonoid, Luteolin, Negatively Affects Neuronal Differentiation

**DOI:** 10.3389/fnmol.2019.00041

**Published:** 2019-03-08

**Authors:** Amrutha Swaminathan, Moumita Basu, Abdelhamid Bekri, Pierre Drapeau, Tapas K. Kundu

**Affiliations:** ^1^Transcription and Disease Laboratory, Molecular Biology and Genetics Unit, Jawaharlal Nehru Centre for Advanced Scientific Research, Bengaluru, India; ^2^Department of Biochemistry, University of Montreal, Montreal, QC, Canada; ^3^Université de Montréal Hospital Research Centre (CRCHUM), Université de Montréal, Montreal, QC, Canada; ^4^Department of Neurosciences, University of Montreal, Montreal, QC, Canada

**Keywords:** flavonoid, embryonic stem cells, neuronal differentiation, lysine acetyltransferase, p300

## Abstract

Luteolin, a polyphenolic plant flavonoid, has been attributed with numerous beneficial properties like anti-cancer, antioxidant, and anti-inflammatory action. Luteolin has been reported earlier to be neuroprotective in models of spinal cord injury and traumatic brain injury and also induces neurite outgrowth in PC12 cells. However, the effect of luteolin on early differentiation, which might be important for its beneficial effects, is unknown. In this report, we show that luteolin negatively affects early differentiation of embryonic stem cells, hampering the formation of embryoid bodies. At later stages of differentiation, luteolin specifically inhibits neuronal differentiation, where the expression of early neuronal markers is suppressed, whereas luteolin treatment does not inhibit expression of meso- and endodermal markers. Further, in a developing zebrafish model, luteolin treatment leads to fewer numbers of mitotic cells in the brain. These specific effects of luteolin on neuronal differentiation could possibly be due to its ability to inhibit the lysine acetyltransferase, p300, since the structurally closely related p300 non-inhibitor flavonoid, apigenin, does not inhibit neuronal differentiation. These results show that luteolin perturbs neuronal differentiation of embryonic stem cells.

## Introduction

Luteolin (3^′^,4^′^,5,7-tetrahydroxyflavone) is a polyphenolic flavone found in many herbs, fruits and vegetables and traditionally used in Chinese medicine, which has been found to have anti-cancer, anti-oxidant, anti-apoptotic, and anti-inflammatory properties (Molnár et al., 1981; Selvi et al., 2015; Xia et al., 2016). However, a detailed study of the mechanisms underlying these beneficial effects is essential to evolve better drugs. Luteolin was reported to also have protective and anti-inflammatory effects in models of traumatic brain injury, spinal cord injury, and MPP+ induced toxicity (Wruck et al., 2007; Paterniti et al., 2013; Xu et al., 2014). While luteolin has been used in brain disease models and in models of injury owing to its ability to cross the blood-brain barrier, it is not yet known whether it does so by affecting the dynamics of pluripotency and differentiation in neural stem cells.

Luteolin has been shown to have neurotrophic activity in PC12 cells (Lin et al., 2010). Luteolin also affects other differentiation pathways like osteoclast differentiation, erythroid, and myeloid differentiation and oligodendrocyte maturation, promoting some pathways and inhibiting others (Lee J.W. et al., 2009; Barbierato et al., 2015; Samet et al., 2015; Skaper et al., 2018). In other reports, it has been shown to maintain pluripotency of periodontal ligament cells by activating Oct4/Sox2 (Liu et al., 2015, 2016). However, it is not known whether luteolin regulates neuronal differentiation, and this knowledge might be important in explaining its positive neuroprotective effect.

Since embryonic stem cells are marked by their unlimited self-renewal capacity and ability to differentiate into almost any cell type, we used it as our primary model system to understand the effect of luteolin on early differentiation. Further, ESCs can differentiate to give EBs, which can simulate the microenvironment of the embryo itself and give rise to endoderm, ectoderm, and mesoderm. Using mouse ESCs, we show here that treatment with luteolin hampers early differentiation and increases expression of pluripotency markers. During later stages of differentiation, luteolin specifically affects neuronal differentiation leading to a reduction in the number of cells differentiating into neurons without inducing apoptosis. We validate this using the developing zebrafish model, where luteolin treatment leads to a reduction in the number of mitotic cells in the brain. Since the closely related flavonoid, apigenin does not lead to the same effects, these effects might be mediated by inhibition of the lysine acetyltransferase, p300, since luteolin does indeed inhibit p300 in embryonic stem cells.

## Materials and Methods

### mESC Culture

The mESC line, E14Tg2a was procured from ATCC. Cells were cultured on tissue culture grade plasticware coated with 0.1% gelatin following manufacturer’s instructions. Briefly, cells were grown in DMEM media containing 10% fetal bovine serum, 1X non-essential amino acids, 1 mM sodium pyruvate, 0.1 mM beta-mercaptoethanol, and 1000 U/ml leukaemia inhibitory factor (LIF) at 37°C. Medium was changed every 24 h. Confluent colonies were split following trypsinization. EBs were set up using 1 × 10^6^ cells seeded into a low-attachment petri plate and incubated for 48 h before observing the EBs. When effect of compounds on early differentiation was studied, EBs were treated from the time of initiation. EBs were observed and imaged every day using the same imaging conditions. Quantification of EB area was performed using ImageJ.

### Retinoic Acid Mediated Differentiation

Retinoic acid was used to induce neural differentiation from EBs. EBs were generated in the presence of 1 μM all-trans retinoic acid for 48 h and 50 EBs were transferred to a 6 cm cell culture treated dish with poly-lysine coated coverslips. Differentiation was monitored in the presence of DMSO or compound for 5 days and the differentiating colonies were used for immunocytochemistry against β-III-tubulin.

For direct differentiation, ES cells were differentiated directly in medium containing 0.5 μM all-trans retinoic acid. Cells were treated with DMSO/luteolin for 24 or 60 h and harvested for qRT-PCR analysis to check the levels of *Sox1* and *Pax6* expression.

### Zebrafish

Routine zebrafish (*Danio rerio*) maintenance was performed according to standard procedures at 28.5°C under 12 h light/12 h dark cycles at the Centre Hospitalier de l’Université de Montréal Research Centre (CRCHUM), Montréal, QC, Canada. All experiments were performed in compliance with the guidelines of the Canadian Council for Animal Care. 8 hpf larvae from transgenic *nestin*: GFP zebrafish (provided by Jana Maier – Karlsruhe Institute of Technology, Germany) in which neural stem cells are fluorescently labeled were used for luteolin treatment and immunostaining. For counting the phosphorylated histone H3S10-positive cells, 10–15 48 hpf larvae were used for immunostaining followed by whole brain imaging.

### Immunocytochemistry

Embryoid bodies differentiated using retinoic acid on poly-lysine coated coverslips were treated for 5 days with DMSO or compound. After the treatment, cells were washed with 1X PBS and fixed in 4% paraformaldehyde for 20 min at room temperature. The cells were permeabilised using 0.5% Triton X-100 for 10 min. Following washes with 1X PBS thrice for 10 min, blocking was performed using 5% FBS in 1X PBS for 45 min at 37°C. Immunostaining was performed using α-beta-III-tubulin (1:200) for 1 h at room temperature. Following three washes with wash buffer (1% FBS in 1X PBS), the cells were incubated with a secondary antibody tagged with a fluorescent dye (Alexa-fluor 568, goat anti-rabbit 1:400) for 1 h at room temperature. After three washes, nuclei were stained with Hoechst (1 μg/ml) for 20 min. Following washes, coverslips were mounted in 70% glycerol and staining of the EBs was observed using Carl Zeiss confocal microscope LSM510 META.

For whole mount staining of zebrafish embryos, zebrafish embryos expressing GFP-tagged Nestin treated with DMSO or luteolin for 40 h from 8 hpf were anaesthesized and fixed in 4% paraformaldehyde overnight at 4°C. Following washes with PBS for an hour, larvae were incubated with 1 mg/ml collagenase for 20 min to remove the skin. After washing the collagenase with 1X PBS, larvae were blocked with 1X PBS containing goat serum, DMSO, and Triton X-100 for 1 h at room temperature and then incubated in blocking solution containing the primary antibody against phosphorylated serine 10 on histone H3 (Millipore, #06-570) overnight at 4°C. After washing with PBST, larvae were incubated in block solution containing secondary fluorescently tagged antibody overnight at 4°C. Similarly, incubation with primary antibody against GFP and secondary antibody were performed in these embryos. After the double staining, larvae were transferred to 70% glycerol and mounted on a slide. The larvae were visualized using a Quorum Technologies spinning disk confocal microscope with a CSU10B spinning head mounted on an Olympus BX61W1 fluorescence microscope and connected to a Hamamatsu ORCA-ER camera. Images from 6 to 8 larval brains were acquired and used for the counting and further analysis of phospho-H3S10-positive cells using Volocity software.

### qRT-PCR

ESC or EBs treated with DMSO or luteolin were lysed using TRIzol. RNA was extracted by chloroform phase separation followed by precipitation with isopropanol and ethanol washes before dissolving in water. cDNA was synthesized using oligo (dT) and Moloney murine leukemia virus reverse transcriptase (Sigma). Expression analysis was performed using SYBR green mix (Bioline SensiFAST SYBR NO-ROX). Primers used for *Sox1*: FP: TCAAACGGCCCATGAACGCCTTC; RP: TCCGGGTGCTCCTTCATGTGC; *Pax6*: FP: AACGATAACATACCAAGCGTGT; RP: GGTCTGCCCGTTCAACATC; *Oct4*: FP: TCCTGGAGGGCCAGGAATCGG; RP: CATCGGAGTTGCTCTCCAC; *Sox17*: FP: CCGATGAACGCTTTCATGGTGTG; RP: CTCCACCCGCTTCAGCCGCTTC; *Tbx2*: FP: CTGCACGTCTCGGCACTGGGCC; RP: CCGCTGACTCGCACCTTGAAGG.

### Western Blot Analysis

E14Tg2a mESCs were treated with 15 μM luteolin for 12 h. Lysates prepared from DMSO treated and luteolin treated cells were run on a 12% SDS-PAGE gel. Following electrophoresis, proteins were electro-transferred from the gel onto PVDF membrane (Millipore). After blocking with 5% non-fat milk solution prepared in 1X PBS and immunoblotted with α-acetylated lysine 9 of histone H3 (H3K9ac), α-acetylated lysine 14 of histone H3 (H3K14ac) or α-histone H3. After washes, detection was performed using goat anti-rabbit horse radish peroxidase conjugated secondary antibody (from Abcam). Bands were visualized using ECL detection system (Bio-Rad).

## Results

### Luteolin Impacts Early Ectodermal Differentiation Into Neural Lineage

Luteolin has been shown earlier to have neuroprotective and other beneficial actions. In order to check if these are brought about by modulation of proliferation and differentiation of stem cells, embryonic stem cells were treated with luteolin while differentiating them to EBs (Supplementary Figure S1A). Embryonic stem cells were chosen for this purpose over other cellular models, since they have a normal genetic background (unlike cell lines of tumor origin from later stages of differentiation or from non-neural tissue of origin). Luteolin treatment led to a remarkable inhibition of embryoid body formation (Figure\ 1A and Supplementary Figure S1B), probably due to inhibition of differentiation.

**FIGURE 1:**
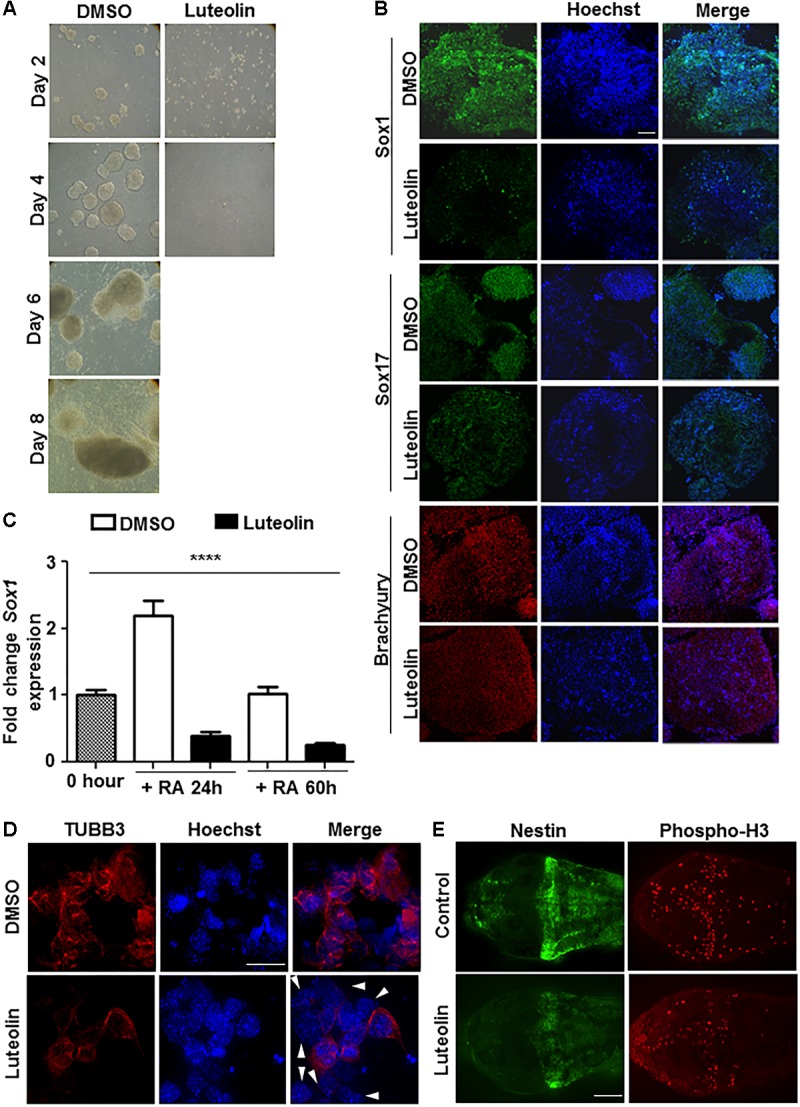
Luteolin treatment perturbs early embryonic stem cell differentiation to the neuronal lineage. **(A)** Bright-field images of EBs treated with DMSO/luteolin acquired every alternate day show that luteolin treatment strongly prevents EB formation, while DMSO treated control mESCs formed steadily growing EBs. Images of luteolin treated cells beyond day 4 are not shown, since they did not grow any further (*N* = 2, *n* = 100 EBs per treatment per time point). **(B)** Immunofluorescence analysis of germline marker expression in EBs treated with DMSO or luteolin (15 μM) for 24 h from 48 h. The expression of Sox1 (ectodermal marker), Sox17 (endodermal marker), and Brachyury (mesodermal marker) were analyzed. Scale bar: 50 μm (*N* = 2, *n* = 3–5 EBs imaged per treatment per marker). **(C)** qRT-PCR analysis of ESCs treated with retinoic acid (0.5 μM) and DMSO or luteolin (15 μM) for 24 and 60 h to quantify expression of early neuronal marker *Sox1*. DMSO treated undifferentiated ESCs were taken as control, GAPDH expression was used for normalization. While the expression of both markers increased with time in DMSO and RA treated ESCs, luteolin treatment led to a significant reduction in the expression of both markers. One-way ANOVA performed for the factor of treatment condition: ^∗∗∗∗^*p* < 0.0001 (*N* = 2, *n* = 3 per treatment). **(D)** Immunofluorescence analysis of neuronal marker expression in 2-day old EBs grown in the presence of 1 μM RA and differentiated further for 5 days with DMSO or luteolin (15 μM). While β-III tubulin was uniformly expressed in all the DMSO treated control differentiating cells, many luteolin treated cells did not express the neuronal marker (indicated using white arrowheads). Scale bar: 10 μm (*N* = 2; *n* ≥ 5 fields per treatment). **(E)** Immunofluorescence analysis of the expression of Nestin (neuronal marker) and phosphorylated histone H3 (marker of proliferating cells) in 48 h post fertilization zebrafish embryos treated with DMSO or luteolin (100 μM) from 8 hpf. Luteolin treatment led to less cell division in the brain and lesser expression of Nestin itself. Scale bar: 40 μm (*N* = 2, *n* = 6–8 zebrafish larval heads per treatment).

In order to assess which differentiation pathways were affected by luteolin treatment, EBs were allowed to form and luteolin treatment was performed from 48 to 72 h after EB initiation. At this stage, the EBs have a definite form, and markers of the three germ layers are expressed. Surprisingly, luteolin treatment had a specific effect on ectodermal differentiation as evidenced by a reduction in the expression of *Sox1*, rather than a broad effect (Figure\ 1B).

Since the neuronal system is a major organ of ectodermal origin, the effect of luteolin treatment on neuronal differentiation was analyzed. Since retinoic acid promotes neural lineage specification, expression of neural markers like *Sox1*, *Pax6*, and beta-III-tubulin upon retinoic acid induced differentiation was quantified. The expression of all these markers of early neuronal differentiation was significantly reduced (Figure\s 1C,D and Supplementary Figure S1C). Notably, this was due to lesser number of cells expressing the markers, rather than a broad downregulation of expression in all cells (Figure\ 1D).

In order to confirm this effect of luteolin on neuronal differentiation *in vivo*, developing zebrafish embryos were subjected to luteolin treatment. Treatment with luteolin for 40 h from 8 h post fertilization (hpf) resulted in lesser number of mitotic cells in the brain as assessed by nestin expression and phospho-H3 staining (Figure\ 1E and Supplementary Figure S1D). These results show that luteolin treatment leads to impaired neuronal differentiation with lesser number of cells differentiating into neurons. However, this effect was specific for neurons, since expression of GFAP was not affected during differentiation (Supplementary Figure S2A).

In order to understand whether the reduction in the number of TUBB3-positive cells (Figure\ 1D) was due to an increase in cell death, MTT assay was performed to quantify cell survival upon luteolin treatment. However, in both embryonic stem cells and in EBs, treatment with luteolin did not lead to differences in cell survival (Supplementary Figures S2B,C).

### Inhibitory Action of Luteolin on Neuronal Differentiation Is Probably Through Inhibition of p300

In order to understand whether the effects of luteolin could also be observed with other flavonoids, the effect of the closely related flavonoid, apigenin on early embryonic stem cell differentiation was assessed (Figure\ 2A). However, apigenin showed minimum inhibition of embryoid body formation in contrast to luteolin (Figure\s 2B,C). Further, upon checking the expression of germline markers, *Sox1* expression was significantly downregulated in the presence of luteolin, but not upon treatment with apigenin (Figure\s 2D,E). The expression of the stemness marker, *Oct4*, was upregulated (even after 48 h of luteolin treatment), as has been observed in an earlier studies (Liu et al., 2015) (Figure\ 2D and Supplementary Figure S2D). These observations were intriguing because luteolin and apigenin differ by only one hydroxyl group. Upon further study, it was reported earlier that this hydroxyl group in luteolin is critical for its specific inhibitory effect on the lysine acetyltransferase, p300 (Selvi et al., 2015). p300 is expressed early during neural development, and absence of p300 leads to neural tube closure defects in mice and embryonic lethality (Yao et al., 1998; Partanen et al., 1999). Acetylation of histone H3 and H4 by p300 have been specifically associated with neuronal differentiation (Teo et al., 2005; Lee S. et al., 2009; Li et al., 2017). Hence, it is possible that the inhibitory effect of luteolin on neuronal differentiation is through inhibition of p300. In order to confirm this, histone acetylation levels in stem cells treated with luteolin and apigenin were analyzed, and a decrease in histone acetylation was observed upon luteolin treatment, but not with apigenin treatment (Figure\ 2F). Hence, the effect of luteolin on neural differentiation could be at least in part attributed to its ability to inhibit p300.

**FIGURE 2:**
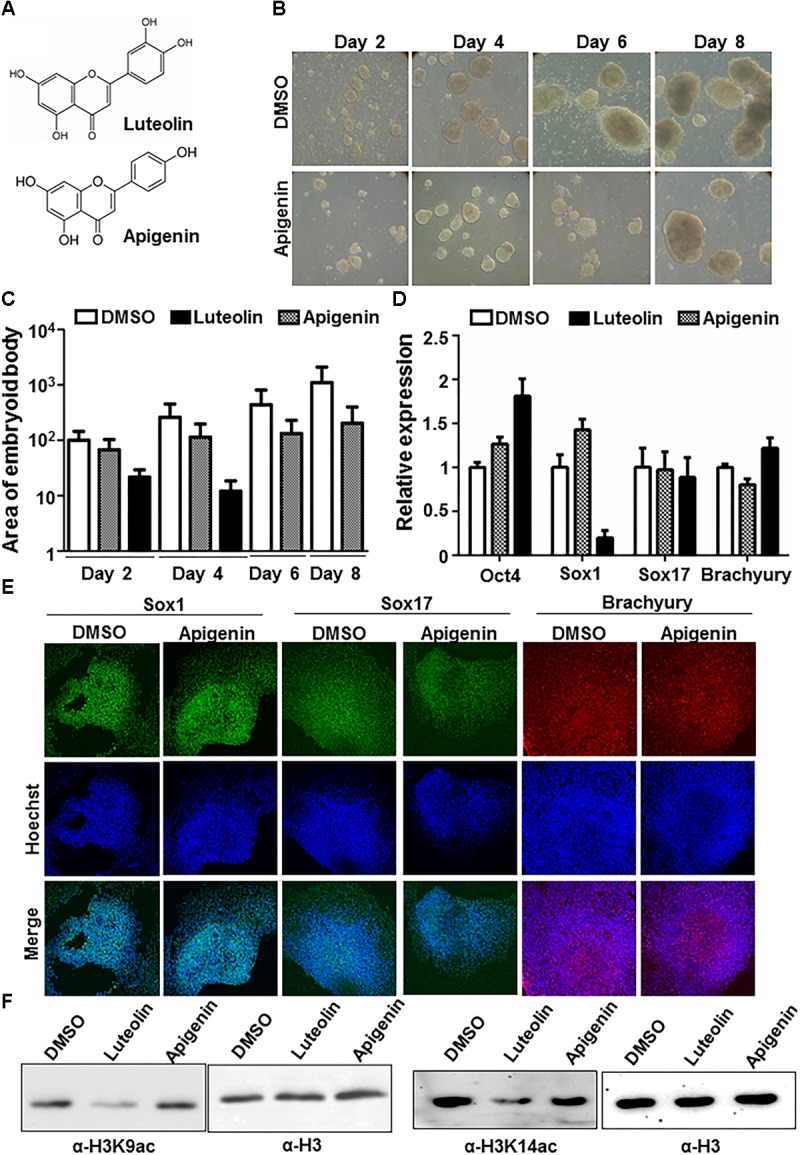
Effect of luteolin on neuronal differentiation could be mediated by p300 inhibition. **(A)** Structure of luteolin and apigenin. Apigenin lacks the hydroxyl group that is critical for the p300-inhibitory potential of luteolin. **(B)** Bright-field images of EBs treated with apigenin acquired every alternate day show that apigenin treated control mESCs formed steadily growing EBs. **(C)** Quantification of (1A and 2B) showing the area of EBs from the 2D images acquired (arbitary units). Note that the scale is exponential (*N* = 2, *n* = 100 per sample per time point). One-way ANOVA with multiple comparisons shows significant increase of DMSO treated EB size from day 2 to day 8, with significant differences in EB size between DMSO and luteolin treated samples from day 4, but not on day 2. **(D)** qRT-PCR analysis of DMSO, luteolin or apigenin treated EBs for germline markers *Sox1, Sox17, Brachyury,* and stemness marker, *Oct4*. DMSO treated EBs were taken as control, GAPDH expression was used for normalization. *N* = 2, *n* = 3 per treatment (One way ANOVA with multiple comparisons for each gene shows significant differences between DMSO and luteolin treatment for *Oct4* and *Sox1* expression, with no difference between DMSO and apigenin). **(E)** Immunofluorescence analysis of germline marker expression in EBs treated with DMSO or apigenin for 24 h from 48 h. The expression of Sox1 (ectodermal marker), Sox17 (endodermal marker), and Brachyury (mesodermal marker) were analyzed. Scale bar: 50 μm. (*N* = 2, *n* = 3–5 EBs imaged per treatment per marker) **(F)** Immunoblotting of lysates extracted from E14Tg2a mESCs using antibodies against histone H3 lysine 9 acetylation, H3 lysine 14 acetylation, and H3. Treatment of mESCs with 15 μM luteolin results in a decrease in H3K9 and H3K14 acetylation in comparison to treatment with DMSO and apigenin treated cells.

## Discussion

Luteolin treatment inhibits early differentiation into EBs and at later stages of differentiation, it specifically affects neuronal differentiation. Luteolin acts positively on some and negatively on some other differentiation pathways. For instance, it stimulates erythroid and myeloid differentiation but inhibits osteoclast differentiation (Lee J.W. et al., 2009; Samet et al., 2015). The results presented here show that it has a negative effect on neuronal differentiation. This could be in part due to its p300 inhibitory action. Histone acetylation and p300 activity have been implicated in neuronal differentiation and neurite formation in multiple studies (Teo et al., 2005; Tomioka et al., 2014; Li et al., 2017). Our results correlate well with an earlier report showing that the lysine acetyltransferase inhibitor, C646 inhibits neurite outgrowth (Tomioka et al., 2014). Though the effects are at different levels, both inhibitors affect neuronal differentiation and morphology. It has also been specifically demonstrated that retinoic acid facilitates p300 recruitment to neurogenin/RAR complex inducing histone acetylation during neuronal differentiation (Lee S. et al., 2009). Since this study has specifically used retinoic acid induced differentiation paradigm, where the role of p300 is significant, the inhibition of neuronal differentiation was stark. It is possible that using other differentiation paradigms might result in different effects of luteolin.

Phosphorylation of p300 by ERK1/2 results in enhanced enzymatic activity (Gusterson et al., 2002), and luteolin suppresses the MAPK signaling pathway at high concentrations (Lu et al., 2017). In order to check if this inhibition of ERK1/2 also occurs upon luteolin treatment in mESCs, and whether this indirectly contributes to p300 inhibition, the effect of luteolin in mESCs was checked. However, no difference in MAPK signaling was observed upon treating mESCs with 15 μM luteolin (Supplementary Figure S2E). Hence, with the currently used concentrations of luteolin, the effect of the molecule on p300 is direct, rather than through MAPK signaling. In support of the direct inhibition of p300 by luteolin, direct binding of luteolin to p300 has been shown in Selvi et al., 2015.

It was also observed that luteolin has no effect on cell survival. However, the detrimental effect of luteolin on neuronal differentiation could also be due to a decrease in cell proliferation. This is supported by an earlier report showing that luteolin treatment leads to G2/M arrest in colon cancer cells, though at higher concentrations than has been used in this study (Chen et al., 2018). Hence, it would be interesting to check whether luteolin indeed affects cell cycle also in stem cells. However, rather than a broad effect, it is possible that luteolin specifically affects cells which are primed into the neuronal lineage, since none of the other germ layer markers were affected.

The flavonoid, luteolin, is a component of many plants, herbs, and medicinal products. It has been attributed with beneficial properties including a neuroprotective role. In the present study, it was observed that, contrary to expectation, luteolin inhibits neuronal differentiation. Surprisingly, these effects are not observed upon apigenin treatment. This is supported by previous studies which find apigenin, a non-p300 inhibitor flavonoid to also have neuroprotective properties (Patil et al., 2014). Hence, the mechanism underlying the neuroprotective function of luteolin could be independent of its inhibitory role on neuronal differentiation.

Luteolin, apigenin and other related flavonoids have been shown to have related effects *in vivo*. However, this study also brings out the differential effect of apigenin and luteolin based on their ability to inhibit p300 by virtue of the presence/absence of a critical hydroxyl group. These results show that it is important to consider the significance of the functional groups in the members of the flavonoid class of compounds while analyzing their physiological effects.

## Author Contributions

AS and TK conceptualized the experiments. AS designed and executed most of the experiments with MB and AB and analyzed the data. AS and TK wrote the manuscript. PD and TK supervised the experiments.

## Conflict of Interest Statement

The authors declare that the research was conducted in the absence of any commercial or financial relationships that could be construed as a potential conflict of interest.

## Abbreviations

EBs: embryoid bodies
mESC: mouse embryonic stem cell
