# Corrigendum: The Dietary Flavonoid, Luteolin, Negatively Affects Neuronal Differentiation

**DOI:** 10.3389/fnmol.2019.00122

**Published:** 2019-05-15

**Authors:** Amrutha Swaminathan, Moumita Basu, Abdelhamid Bekri, Pierre Drapeau, Tapas K. Kundu

**Affiliations:** ^1^Transcription and Disease Laboratory, Molecular Biology and Genetics Unit, Jawaharlal Nehru Centre for Advanced Scientific Research, Bengaluru, India; ^2^Department of Biochemistry, University of Montreal, Montreal, QC, Canada; ^3^Université de Montréal Hospital Research Centre (CRCHUM), Université de Montréal, Montreal, QC, Canada; ^4^Department of Neurosciences, University of Montreal, Montreal, QC, Canada

**Keywords:** flavonoid, embryonic stem cells, neuronal differentiation, lysine acetyltransferase, p300

In the original article, we neglected to include the Life Science Education and Training at JNCASR grant supported by the Department of Biotechnology (Sanction no. DBT/INF/22/SP27679/2018) dated 29/09/2018.

A correction has therefore been made to the **Funding** section:

“This work was supported by the Jawaharlal Nehru Centre for Advanced Scientific Research (JNCASR), the Sir JC Bose Fellowship (to TK), Department of Science and Technology (DST), India (Grant No. SR/S2/JCB-28/2010), a grant from the Canadian Institutes of Health Research (to PD), and the Life Science Education and Training at JNCASR grant, supported by the Department of Biotechnology (Sanction no. DBT/INF/22/SP27679/2018) dated 29/09/2018. AS was, and MB is currently supported by Council of Scientific and Industrial Research (CSIR), India.”

The authors apologize for this error and state that this does not change the scientific conclusions of the article in any way. The original article has been updated.

