# Supplementary material for: The Dietary Flavonoid, Luteolin, Negatively Affects Neuronal Differentiation
Source: Front Mol Neurosci. 2019 Mar 8;12:41. doi: 10.3389/fnmol.2019.00041 (PMC6418693; doi:10.3389/fnmol.2019.00041)
Supplement: Supplementary file 1 [file Presentation_1.pdf]

The Dietary Flavonoid, Luteolin, Negatively Affects Neuronal Differentiation

Amrutha Swaminathan[1,2¶#], Moumita Basu[1#], Abdelhamid Bekri[2,4], Pierre Drapeau[3,4] and Tapas K Kundu [1*]

[1] Transcription and Disease Laboratory, Molecular Biology and Genetics Unit, Jawaharlal Nehru Centre for Advanced Scientific Research (JNCASR), Bangalore, India

[2] Department of Biochemistry, Université de Montréal, Montréal, Canada

[3] Department of Neurosciences, Université de Montréal, Montréal, Canada

[4] Research Center of the University of Montréal Hospital Center (CRCHUM), Université de Montréal, Montréal, Canada

[¶] **Present Address:** Department of Molecular Cell Biology, Weizmann Institute of Science, Rehovot, Israel.

[#] Equal contribution

*** Address for correspondence:**

Prof. Tapas K Kundu, Transcription and Disease Laboratory, Molecular Biology and Genetics Unit, Bangalore, India

tapas@jncasr.ac.in

Supplementary figures:

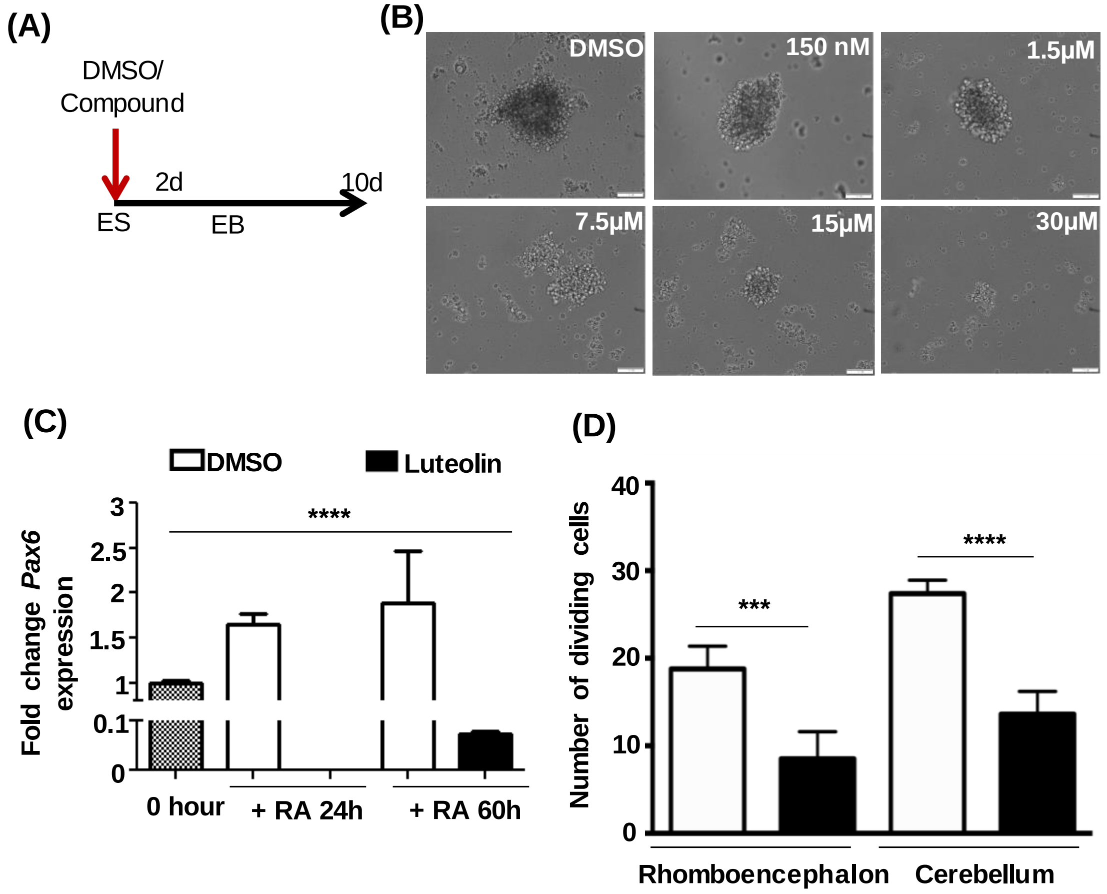

Figure S1: (A) Scheme showing the timeline of treatment followed in 1(A) and 2(B). (B) Bright field images of EBs treated with DMSO/luteolin after 36 hours of induction show that luteolin treatment has a dose-dependent effect on EB formation. Scale: 100 µm. (C) qRT-PCR analysis of ESCs treated with retinoic acid (0.5 µM) and DMSO or luteolin (15 µM) for 24 and 60 hours to quantify expression of early neuronal markers *Pax6.* DMSO treated undifferentiated ESCs were taken as control, GAPDH expression was used for normalization. One-way ANOVA: $p<0.0001$. (D) Quantification of the number of dividing cells in 2 brain regions showing the significant reduction upon luteolin treatment (n=6 embryos per treatment). Unpaired t-test of luteolin vs DMSO: Rhomboencephalon:$p<0.001$; Cerebellum:$p<0.0001$.

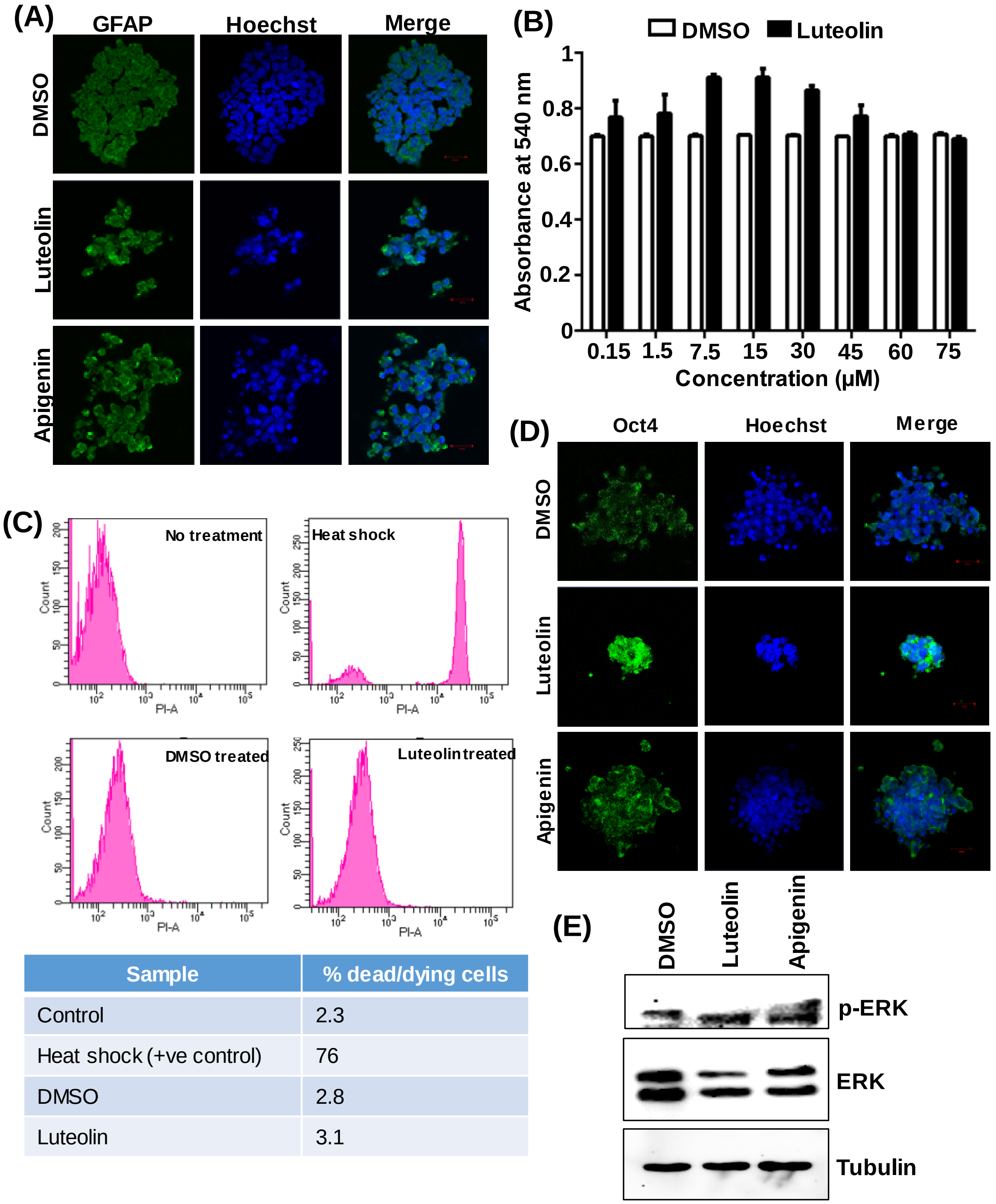

Sample	% dead/dying cells
Control	2.3
Heat shock (+ve control)	76
DMSO	2.8
Luteolin	3.1

Figure S2: (A) Immunofluorescence analysis of glial marker expression GFAP in EBs treated with DMSO, luteolin or apigenin (15 μM) for 24 hours from 48 hours. Scale bar: 20 μm. (B) MTT assay of mESCs and EBs treated with DMSO, luteolin or apigenin for 24 hours. (C) Flow cytometric analysis of E14Tg2a cells treated with DMSO/luteolin/heat shock, washed, fixed and

stained with PI. (D) Immunofluorescence analysis of the pluripotency marker expression Oct4 in EBs treated with DMSO, luteolin or apigenin (15 μM) for 24 hours from 48 hours. Scale bar: 20 μm. (E) Immunoblotting of lysates extracted from E14Tg2a mouse embryonic stem cells using antibodies against phospho-ERK, ERK and tubulin. Treatment of mESCs with 15 μM luteolin or apigenin did not result in any change in phospho-ERK levels in comparison to treatment with DMSO treated cells.

Supplementary methods:

MTT assay:

Equal number of E14Tg2a cells (~ $3x10^4$ cells/well) or embryoid bodies were seeded into a 96-well plate and treated with 15μM compound/DMSO for 24 hours in 100μL of respective supplemented media. Post-treatment, 10μL of 5mg/mL MTT reagent (Sigma, Cat# M5655 dissolved in 1X PBS) was added and incubated at 37°C for 3 hours in 90μL serum free media. MTT-formazan was dissolved in 100μL DMSO by incubating at 37°C for another 2 hours after discarding the media; absorbance at 540nm was measured using VERSA max microplate reader (Molecular Devices).

Flow cytometry analysis:

For FACS, mESCs were grown to ~80-90% confluency and treated with 15uM luteolin and equal vol (5uL) of DMSO for 24 hours. Cells were harvested by trypsinization and collected by centrifugation. Cells were resuspended in 1X annexin binding buffer (BD Biosciences kit 556547) by maintaining total population of 1-2 X 10^6 cells/mL. 100 μL cell suspension was incubated with 5 μL Annexin V FITC and 5 μL PI (BD Biosciences- kit cat # 556547) for 10 minutes in dark at RT. Cells were flushed with 400 μL of 1X annexin V binding buffer to stop the reaction.

Unstained, single (FITC or PI) stained, heat treated cells were used as controls. Cells were analyzed by BD FACSDiva 8.0.1 flow cytometer (70 μm nozzle).
